# 2-(2-Nitro­phen­yl)-1,3-dioxan-5-ol

**DOI:** 10.1107/S1600536809043402

**Published:** 2009-10-28

**Authors:** Jin Chen, Xukang Ren, Zhaobo Li, Xiaohua Du

**Affiliations:** a State Key Laboratory Breeding Base of Green Chemistry-Synthesis Technology, Zhejiang University of Technology, Hangzhou 310014, People’s Republic of China

## Abstract

In the title compound, C_10_H_11_NO_5_, the six-membered 1,3-dioxane ring displays a chair conformation, with the hydr­oxy and 2-nitro­phenyl groups in equatorial positions, which minimizes steric hindrance. In the crystal, mol­ecules are linked into chains along the *b* axis by inter­molecular O—H⋯O hydrogen bonds.

## Related literature

For background to the condensation of glycerol with aldehydes and ketones to [1,3]dioxan-5-ols and [1,3]dioxolan-4-yl-methanols, see: Deutsch *et al.* (2007 ▶); Hill *et al.* (1928 ▶). Six-membered ring acetals are potential precursors for the production of the green platform chemicals, e.g. 1,3-dihydroxy­acetone and 1,3-propane­diol, see: Wang *et al.* (2003 ▶, 2009 ▶). For a related structure, see: Li *et al.* (2009 ▶).
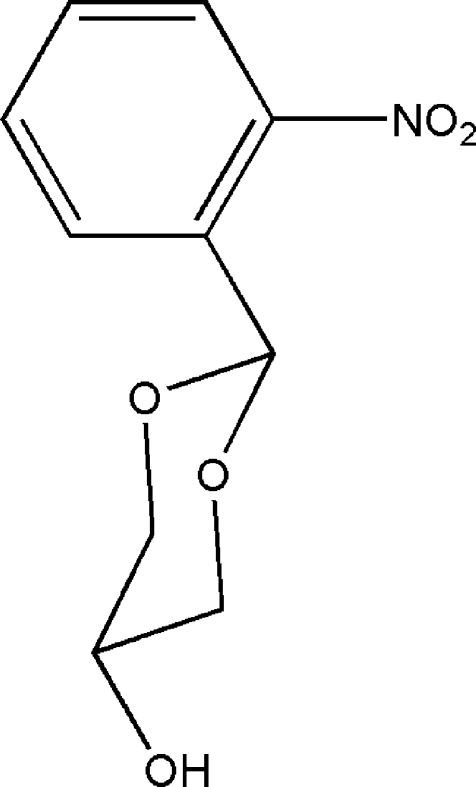


## Experimental

### 

#### Crystal data

C_10_H_11_NO_5_
*M* *_r_* = 225.20
Monoclinic, 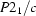

*a* = 8.0166 (4) Å
*b* = 10.6499 (5) Å
*c* = 12.4109 (6) Å
β = 101.221 (1)°
*V* = 1039.34 (9) Å^3^
*Z* = 4
Mo *K*α radiation
μ = 0.12 mm^−1^
*T* = 296 K
0.35 × 0.19 × 0.12 mm

#### Data collection

Rigaku R-AXIS RAPID diffractometer
Absorption correction: multi-scan (*ABSCOR*; Higashi, 1995 ▶) *T*_min_ = 0.960, *T*_max_ = 0.986
9906 measured reflections
2346 independent reflections
1466 reflections with *I* > 2σ(*I*)
*R*_int_ = 0.025

#### Refinement

*R*[*F* ^2^ > 2σ(*F* ^2^)] = 0.041
*wR*(*F* ^2^) = 0.110
*S* = 1.00
2346 reflections
147 parameters
H-atom parameters constrained
Δρ_max_ = 0.18 e Å^−3^
Δρ_min_ = −0.17 e Å^−3^

### 

Data collection: *PROCESS-AUTO* (Rigaku, 2006 ▶); cell refinement: *PROCESS-AUTO*; data reduction: *CrystalStructure* (Rigaku/MSC, 2007 ▶); program(s) used to solve structure: *SHELXS97* (Sheldrick, 2008 ▶); program(s) used to refine structure: *SHELXL97* (Sheldrick, 2008 ▶); molecular graphics: *ORTEP-3 for Windows* (Farrugia, 1997 ▶); software used to prepare material for publication: *WinGX* (Farrugia, 1999 ▶).

## Supplementary Material

Crystal structure: contains datablocks global, I. DOI: 10.1107/S1600536809043402/pv2219sup1.cif

Structure factors: contains datablocks I. DOI: 10.1107/S1600536809043402/pv2219Isup2.hkl

Additional supplementary materials:  crystallographic information; 3D view; checkCIF report

## Figures and Tables

**Table 1:** Hydrogen-bond geometry (Å, °)

*D*—H⋯*A*	*D*—H	H⋯*A*	*D*⋯*A*	*D*—H⋯*A*
O1—H101⋯O3^i^	0.82	2.08	2.8548 (18)	157

Symmetry code: (i) 
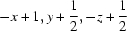
.

**Fig. 1.**
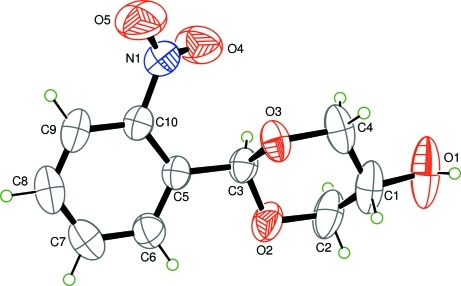
The asymmetric unit of the structure of the title compound, with the atomic labeling scheme. Displacement ellipsoids are drawn at the 50% probability level.

**Fig. 2.**
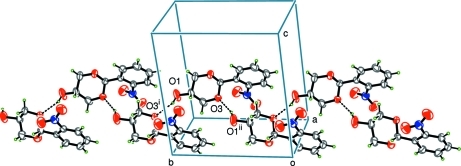
Unit cell packing of the title compound showing H-bonded chains of molecules lying along the b-axis.

## supplementary crystallographic information

### Comment

The condensation of glycerol, a renewable raw materials, with aldehydes and ketones to [1,3]dioxan-5-ols and [1,3]dioxolan-4-yl-methanols was investigated for many years (Hill *et al.*, 1928; Deutsch *et al.*, 2007). The condensation products are used widely as novel chemical intermediates. The six-membered ring acetals are potential precursors for the production of the green platform chemicals, e.g., 1,3-dihydroxyacetone (Wang *et al.*, 2009) and 1,3-propanediol (Wang *et al.*, 2003).

In this article, we report the crystal structure of the title compoud (Fig. 1) which has been determined in our laboratory. Its main structure unit is a six-membered ring, [1,3]dioxan, which displays a chair conformation, with the hydroxyl and the 2-nitrophenyl groups in the equatorial positions. The atoms C1 and C3 of the [1,3]dioxan ring lie 0.635 (2) and 0.682 (3)Å, respectively, from the mean plane of O2/O3/C4/C2. The dihedral angel between the mean plane O2/O3/C4/C2 and the benzene ring is 32.12(14 °. The molecules are linked into chains along the *b*-axis involving intermolecular hydrogen bond of the type O—H···O (Table 1) thus stabilizing the crystal structure (Fig. 2).

### Experimental

The title compound was synthesized by treating glycerol (2.76 g, 30 mmol) with 2-nitrobenzaldehyde (3.02 g, 20 mmol) in the presence of *p*-toluenesulfonic (0.06 g) as a catalyst in cyclohexane (40 ml). This reaction mixture was placed in a two-necked round-bottomed flask fitted with a magnetic stirrer and Dean-Stark assembly. The mixture was refluxed with stirring and water present in the reaction was removed as an azeotropeover for 6 h. Once the reaction was complete, the mixture was washed with water, and the solvent was distilled under vacuum. The resulting reaction mixture was purified directly by silica gel column chromatography (eluent:petroleum ether/EtOAc; 7:4). Single crystals were obtained by slow evaporation of acetone/hexane mixture (1:1) of the title compound.

### Refinement

H atoms were placed in calculated position with C—H = 0.98, 0.97  and 0.93 Å for methine, methylene and aryl H-atoms, respectively, and O—H = 0.82 Å. All H atoms were refined in riding mode, with *U*_iso_(H) = 1.2*U*_eq_ of the carrier atoms.

### Crystal data

**Table:**

C_10_H_11_NO_5_	*F*(000) = 472
*M**_r_* = 225.20	*D*_x_ = 1.439 Mg m^−^^3^
Monoclinic, *P*2_1_/*c*	Mo *K*α radiation, λ = 0.71073 Å
Hall symbol: -P 2ybc	Cell parameters from 6214 reflections
*a* = 8.0166 (4) Å	θ = 3.2–27.4°
*b* = 10.6499 (5) Å	µ = 0.12 mm^−^^1^
*c* = 12.4109 (6) Å	*T* = 296 K
β = 101.221 (1)°	Chunk, colorless
*V* = 1039.34 (9) Å^3^	0.35 × 0.19 × 0.12 mm
*Z* = 4	

### Data collection

**Table:**

Rigaku R-AXIS RAPID diffractometer	2346 independent reflections
Radiation source: rolling anode	1466 reflections with *I* > 2σ(*I*)
graphite	*R*_int_ = 0.025
Detector resolution: 10.00 pixels mm^-1^	θ_max_ = 27.4°, θ_min_ = 3.2°
ω scans	*h* = −9→10
Absorption correction: multi-scan (*ABSCOR*; Higashi, 1995)	*k* = −13→13
*T*_min_ = 0.960, *T*_max_ = 0.986	*l* = −16→15
9906 measured reflections	

### Refinement

**Table:**

Refinement on *F*^2^	Secondary atom site location: difference Fourier map
Least-squares matrix: full	Hydrogen site location: inferred from neighbouring sites
*R*[*F*^2^ > 2σ(*F*^2^)] = 0.041	H-atom parameters constrained
*wR*(*F*^2^) = 0.110	*w* = 1/[σ^2^(*F*_o_^2^) + (0.0372*P*)^2^ + 0.330*P*] where *P* = (*F*_o_^2^ + 2*F*_c_^2^)/3
*S* = 1.00	(Δ/σ)_max_ < 0.001
2346 reflections	Δρ_max_ = 0.18 e Å^−^^3^
147 parameters	Δρ_min_ = −0.17 e Å^−^^3^
0 restraints	Extinction correction: *SHELXL97* (Sheldrick, 2008), Fc^*^=kFc[1+0.001xFc^2^λ^3^/sin(2θ)]^-1/4^
Primary atom site location: structure-invariant direct methods	Extinction coefficient: 0.0152 (19)

### Special details

**Table:**

Geometry. All e.s.d.'s (except the e.s.d. in the dihedral angle between two l.s. planes) are estimated using the full covariance matrix. The cell e.s.d.'s are taken into account individually in the estimation of e.s.d.'s in distances, angles and torsion angles; correlations between e.s.d.'s in cell parameters are only used when they are defined by crystal symmetry. An approximate (isotropic) treatment of cell e.s.d.'s is used for estimating e.s.d.'s involving l.s. planes.
Refinement. Refinement of *F*^2^ against ALL reflections. The weighted *R*-factor *wR* and goodness of fit *S* are based on *F*^2^, conventional *R*-factors *R* are based on *F*, with *F* set to zero for negative *F*^2^. The threshold expression of *F*^2^ > σ(*F*^2^) is used only for calculating *R*-factors(gt) *etc*. and is not relevant to the choice of reflections for refinement. *R*-factors based on *F*^2^ are statistically about twice as large as those based on *F*, and *R*- factors based on ALL data will be even larger.

### Fractional atomic coordinates and isotropic or equivalent isotropic displacement parameters (Å^2^)

**Table:**

	*x*	*y*	*z*	*U*_iso_*/*U*_eq_	
O3	0.65238 (15)	0.68340 (10)	0.28615 (9)	0.0558 (3)	
C5	0.78112 (19)	0.53115 (14)	0.41276 (12)	0.0456 (4)	
O2	0.70918 (18)	0.73226 (11)	0.47090 (10)	0.0693 (4)	
N1	0.89732 (19)	0.48237 (15)	0.24515 (13)	0.0616 (4)	
C9	0.8397 (2)	0.31608 (16)	0.36545 (16)	0.0632 (5)	
H9	0.8762	0.2600	0.3174	0.076*	
C3	0.7680 (2)	0.66933 (14)	0.38712 (13)	0.0485 (4)	
H3	0.8797	0.7022	0.3804	0.058*	
C10	0.83678 (19)	0.44328 (15)	0.34425 (13)	0.0488 (4)	
C6	0.7294 (2)	0.48360 (17)	0.50467 (14)	0.0588 (5)	
H6	0.6903	0.5386	0.5524	0.071*	
C8	0.7884 (3)	0.27296 (19)	0.45772 (18)	0.0716 (6)	
H8	0.7905	0.1874	0.4730	0.086*	
O4	0.9752 (2)	0.57938 (16)	0.24619 (14)	0.0940 (5)	
O1	0.6154 (2)	1.02233 (13)	0.3183 (2)	0.1241 (8)	
H101	0.5292	1.0497	0.2787	0.149*	
C2	0.7052 (3)	0.86500 (18)	0.45027 (19)	0.0837 (7)	
H2A	0.8195	0.8949	0.4502	0.100*	
H2B	0.6628	0.9084	0.5082	0.100*	
C1	0.5922 (3)	0.89338 (17)	0.3408 (2)	0.0783 (7)	
H1	0.4730	0.8775	0.3446	0.094*	
C4	0.6432 (3)	0.81319 (17)	0.25365 (17)	0.0706 (6)	
H4A	0.5610	0.8228	0.1856	0.085*	
H4B	0.7531	0.8403	0.2408	0.085*	
C7	0.7342 (3)	0.3565 (2)	0.52745 (16)	0.0700 (5)	
H7	0.7004	0.3274	0.5905	0.084*	
O5	0.8708 (2)	0.41251 (17)	0.16609 (13)	0.1034 (6)	

### Atomic displacement parameters (Å^2^)

**Table:**

	*U*^11^	*U*^22^	*U*^33^	*U*^12^	*U*^13^	*U*^23^
O3	0.0629 (7)	0.0404 (6)	0.0568 (7)	−0.0064 (5)	−0.0067 (5)	0.0055 (5)
C5	0.0449 (8)	0.0405 (9)	0.0484 (8)	0.0015 (6)	0.0018 (7)	−0.0013 (7)
O2	0.0931 (9)	0.0483 (7)	0.0641 (8)	0.0167 (6)	0.0093 (7)	−0.0112 (6)
N1	0.0601 (9)	0.0607 (10)	0.0658 (10)	0.0037 (7)	0.0165 (7)	−0.0066 (8)
C9	0.0663 (11)	0.0415 (10)	0.0760 (12)	0.0095 (8)	−0.0007 (9)	−0.0071 (9)
C3	0.0503 (9)	0.0401 (9)	0.0515 (9)	0.0013 (7)	0.0008 (7)	−0.0043 (7)
C10	0.0465 (8)	0.0448 (9)	0.0529 (9)	0.0027 (7)	0.0044 (7)	−0.0020 (7)
C6	0.0661 (11)	0.0551 (11)	0.0547 (10)	0.0040 (8)	0.0102 (8)	0.0037 (8)
C8	0.0802 (13)	0.0446 (11)	0.0809 (13)	−0.0019 (9)	−0.0068 (11)	0.0126 (10)
O4	0.1066 (12)	0.0895 (12)	0.0969 (11)	−0.0285 (10)	0.0472 (9)	−0.0076 (9)
O1	0.0894 (11)	0.0398 (9)	0.214 (2)	0.0013 (7)	−0.0431 (13)	0.0144 (10)
C2	0.0989 (16)	0.0438 (11)	0.0996 (16)	0.0156 (10)	−0.0026 (13)	−0.0185 (10)
C1	0.0624 (11)	0.0373 (10)	0.1237 (18)	0.0046 (8)	−0.0106 (12)	0.0036 (11)
C4	0.0676 (12)	0.0457 (10)	0.0879 (14)	−0.0100 (8)	−0.0114 (10)	0.0209 (10)
C7	0.0750 (13)	0.0679 (13)	0.0638 (11)	−0.0063 (10)	0.0057 (10)	0.0205 (10)
O5	0.1424 (15)	0.1019 (13)	0.0717 (9)	−0.0136 (11)	0.0352 (10)	−0.0322 (9)

### Geometric parameters (Å, °)

**Table:**

O3—C3	1.4142 (18)	C6—C7	1.382 (3)
O3—C4	1.438 (2)	C6—H6	0.9300
C5—C6	1.383 (2)	C8—C7	1.369 (3)
C5—C10	1.395 (2)	C8—H8	0.9300
C5—C3	1.505 (2)	O1—C1	1.421 (2)
O2—C3	1.394 (2)	O1—H101	0.8200
O2—C2	1.436 (2)	C2—C1	1.510 (3)
N1—O4	1.206 (2)	C2—H2A	0.9700
N1—O5	1.2167 (19)	C2—H2B	0.9700
N1—C10	1.468 (2)	C1—C4	1.496 (3)
C9—C8	1.369 (3)	C1—H1	0.9800
C9—C10	1.379 (2)	C4—H4A	0.9700
C9—H9	0.9300	C4—H4B	0.9700
C3—H3	0.9800	C7—H7	0.9300
			
C3—O3—C4	109.84 (12)	C9—C8—H8	120.2
C6—C5—C10	116.10 (15)	C7—C8—H8	120.2
C6—C5—C3	120.83 (15)	C1—O1—H101	109.5
C10—C5—C3	123.00 (15)	O2—C2—C1	110.25 (16)
C3—O2—C2	109.83 (15)	O2—C2—H2A	109.6
O4—N1—O5	122.79 (18)	C1—C2—H2A	109.6
O4—N1—C10	119.27 (15)	O2—C2—H2B	109.6
O5—N1—C10	117.90 (16)	C1—C2—H2B	109.6
C8—C9—C10	119.50 (18)	H2A—C2—H2B	108.1
C8—C9—H9	120.3	O1—C1—C4	110.2 (2)
C10—C9—H9	120.3	O1—C1—C2	106.94 (17)
O2—C3—O3	110.58 (13)	C4—C1—C2	109.60 (16)
O2—C3—C5	109.41 (14)	O1—C1—H1	110.0
O3—C3—C5	107.32 (12)	C4—C1—H1	110.0
O2—C3—H3	109.8	C2—C1—H1	110.0
O3—C3—H3	109.8	O3—C4—C1	110.63 (17)
C5—C3—H3	109.8	O3—C4—H4A	109.5
C9—C10—C5	122.56 (16)	C1—C4—H4A	109.5
C9—C10—N1	116.26 (16)	O3—C4—H4B	109.5
C5—C10—N1	121.19 (15)	C1—C4—H4B	109.5
C7—C6—C5	121.76 (18)	H4A—C4—H4B	108.1
C7—C6—H6	119.1	C8—C7—C6	120.43 (19)
C5—C6—H6	119.1	C8—C7—H7	119.8
C9—C8—C7	119.65 (18)	C6—C7—H7	119.8
			
C2—O2—C3—O3	−65.20 (18)	O5—N1—C10—C9	33.6 (2)
C2—O2—C3—C5	176.81 (14)	O4—N1—C10—C5	35.4 (2)
C4—O3—C3—O2	64.54 (18)	O5—N1—C10—C5	−146.86 (17)
C4—O3—C3—C5	−176.20 (14)	C10—C5—C6—C7	0.5 (2)
C6—C5—C3—O2	3.4 (2)	C3—C5—C6—C7	177.47 (16)
C10—C5—C3—O2	−179.88 (14)	C10—C9—C8—C7	0.3 (3)
C6—C5—C3—O3	−116.59 (16)	C3—O2—C2—C1	58.5 (2)
C10—C5—C3—O3	60.11 (19)	O2—C2—C1—O1	−171.0 (2)
C8—C9—C10—C5	−0.9 (3)	O2—C2—C1—C4	−51.6 (2)
C8—C9—C10—N1	178.63 (16)	C3—O3—C4—C1	−57.32 (19)
C6—C5—C10—C9	0.4 (2)	O1—C1—C4—O3	168.64 (14)
C3—C5—C10—C9	−176.42 (15)	C2—C1—C4—O3	51.2 (2)
C6—C5—C10—N1	−179.05 (14)	C9—C8—C7—C6	0.6 (3)
C3—C5—C10—N1	4.1 (2)	C5—C6—C7—C8	−1.1 (3)
O4—N1—C10—C9	−144.09 (18)		

### Hydrogen-bond geometry (Å, °)

**Table:**

*D*—H···*A*	*D*—H	H···*A*	*D*···*A*	*D*—H···*A*
O1—H101···O3^i^	0.82	2.08	2.8548 (18)	157

Symmetry codes: (i) −*x*+1, *y*+1/2, −*z*+1/2.
